# *Fusarium pseudonygamai* Promotes Blastospore Transformation in *Ophiocordyceps sinensis*: Insights into Microbial Interaction and Key Mechanisms

**DOI:** 10.3390/jof11100746

**Published:** 2025-10-18

**Authors:** Muhammad Zaryab Khalid, Xuehong Zheng, Richou Han, Li Cao

**Affiliations:** Guangdong Key Laboratory of Animal Conservation and Resource Utilization, Guangdong Public Laboratory of Wild Animal Conservation and Utilization, Institute of Zoology, Guangdong Academy of Sciences, Guangzhou 510260, China; zaryabkhalid0003@hotmail.com (M.Z.K.); zxh1234560716@163.com (X.Z.); hanrc@giz.gd.cn (R.H.)

**Keywords:** *Ophiocordyceps sinensis*, *Fusarium pseudonygamai*, transcriptomics, chromatography, mannitol

## Abstract

Chinese cordyceps, a highly valued traditional medicine, is formed when the fungal parasite *Ophiocordyceps sinensis* infects an underground caterpillar (*Thitarodes*). This interaction progresses slowly, as the larvae have a long developmental period and the fungus requires several months to complete its growth. The medicinal fungus *O. sinensis* has a complex life cycle that requires successful transformation from blastospores to hyphae for the formation of Chinese cordyceps. Building on our previous identification of diverse microbial communities associated with *Thitarodes xiaojinensis* larvae, this study investigates the role of host microbiota in enhancing *O. sinensis* blastospore transformation under in vitro conditions. Cultured supernatant of *Fusarium pseudonygamai* associated with *T. xiaojinensis* larvae significantly increased transformation rates by 31.6% after 8 days. Transcriptomic profiling revealed early upregulation of genes involved in energy metabolism, stress response, detoxification, and hyphal morphogenesis (notably *CYC1*, *hmp*, *gedE*, and *fahA*), supporting the cellular reprogramming required for fungal development. Additionally, mannitol isolated from *F. pseudonygamai* culture acted as a key promoter of transformation. Further functional assays confirmed that modulation of mannitol biosynthesis genes (*M1PDH* and *MDH*) through chemical agonists and inhibitors directly influenced mannitol levels and transformation efficiency. Collectively, these results highlight the pivotal role of microbiota-derived metabolites, particularly mannitol, in regulating *O. sinensis* transformation, offering potential strategies to improve artificial cultivation of Chinese cordyceps.

## 1. Introduction

Chinese cordyceps is a parasitic complex of fungus (*Ophiocordyceps sinensis*) and caterpillar (*Thitarodes*), which has been used for medicinal purposes with a long history in China and other East Asiat countries [1]. *O. sinensis* is endemic to the alpine meadow ecosystems, characterized by low soil temperatures, fragile plant communities, and extreme sensitivity to climate change [2,3]. Additionally, the larvae of *Thitarodes* have a long developmental period. Due to the slow growth rate of *O. sinensis*, it takes between 5 and more than 12 months for the fungus to ultimately kill the host larvae [1,4,5]. Nearly 96.5% of Chinese Cordyceps habitats are located in China, with Qinghai and Xizang accounting for approximately 71.4% of the global harvest [6]. Due to its significant medicinal value, limited distribution, and over-exploitation, natural Chinese cordyceps is exceedingly expensive and cannot meet market demand [7]. Therefore, artificial production is needed to protect natural resources while meeting human consumption demands.

Significant progress has been made in the artificial cultivation of Chinese cordyceps [8,9,10,11]. A critical step in this process is the transformation of blastospores into hyphae, which is necessary for mummifying the infected *Thitarodes* larvae [1,12]. Several factors influence this transformation process, with the microbiota of ghost moth larvae probably playing a crucial role by either supporting or inhibiting *O. sinensis* colonization and growth [13]. Soil microbiota exhibited greater diversity, suggesting that environmental microbial factors might also play a substantial role in the natural occurrence and development of Chinese cordyceps [14].

Our previous study has revealed complex bacterial and fungal communities inhabiting the gut and hemolymph of *Thitarodes* larvae, with certain microbiota hypothesized to increase the blastospores to hyphae transformation [15]. Notably, a recent in vivo study demonstrated that mannitol, a metabolite produced by the fungus, accumulates during the mummification of *T. xiaojinensis* larvae infected by *O. sinensis* and was shown to accelerate this process [16]. This highlights the potential for specific microbiota-derived metabolites to actively promote fungal development and host manipulation. Additionally, based on our bioassay results from about 100 microbial isolates (unpublished data), *F. pseudonygamai* was selected for further evaluation of its influence on *O. sinensis* transformation. To gain deeper insights into the underlying mechanisms, transcriptomic analysis was conducted following *F. pseudonygamai* supernatant treatment. Bioactive metabolites from its crude extracts were also isolated and identified, and their roles in promoting blastospore-to-hyphae transformation were evaluated. Among them, mannitol was identified as a key metabolite promoting the blastospore-to-hyphae transition. Further experiments involving chemical modulation of mannitol biosynthesis genes, specifically *mannitol dehydrogenase* (*MDH*) and *mannitol-1-phosphate dehydrogenase* (*M1PDH*), using NaCl as an agonist and 3-nitrophenyl disulfide as an inhibitor of *M1PDH* [17,18,19], confirmed its functional role. These findings provide valuable insights into the microbial regulation of *O. sinensis* development and suggest potential strategies for improving the artificial cultivation of Chinese cordyceps.

## 2. Materials and Methods

### 2.1. Fungal Strains and Culture Media

The *O. sinensis* isolate KD1223, sourced from wild fruiting bodies in Sichuan, China, was cultured on PPDA (potato dextrose agar supplemented with 10% peptone) medium at 13 °C. The isolate was identified as *O. sinensis* using the ITS region of nuclear ribosomal DNA [9] and preserved at −80 °C at the Guangdong Institute of Applied Biological Resources. First, *O. sinensis* was cultured on PPDA plates at 13 °C for 60 days. Subsequently, the colonies were transferred to liquid PM medium and incubated at 13 °C with shaking at 120 rpm for 15–25 days [15]. The blastospores were harvested by filtering through three layers of sterile lens paper to remove hyphae and large particles, followed by centrifugation at 8000 rpm for 15 min at 10 °C. The supernatant was discarded, and spores were resuspended in phosphate-buffered saline (PBS; pH 6.2) at a final concentration of 2 × 10^8^ blastospores per mL.

*F. pseudonygamai* was identified using the ITS region of nuclear ribosomal DNA and was maintained in the laboratory. It was first cultured on Potato dextrose agar (PDA; 200 g peeled potatoes, 20 g glucose (Fuchen Chemical Reagent Co., Ltd., Tianjin, China, CAS No. 14431-43-7), 15 g agar (Saiguo Biotechnology Co., Ltd., Guangzhou, China, CAS No: 9002-18-0), and 1000 mL water) plates for 3–4 days at 25 °C. *F. pseudonygamai* was then transferred to 250 mL flasks containing 100 mL of Potato dextrose broth (PDB) and incubated with shaking at 120 rpm for additional 3–4 days at 25 °C. The culture was subsequently centrifuged at 8000 rpm for 15 min at 10 °C and the supernatant was filtered using a 0.22 µm filter (Millex^®^-GP, Merck Millipore Ltd., Cork, Ireland). The resulting fungal supernatant was used for further experiments.

### 2.2. Bioassay to Evaluate the Effect of F. pseudonygamai Supernatant on the Transformation of O. sinensis

Bioassay was carried out in a sterile 96-well microtiter plate. Briefly, 90 μL of PM medium (200 g peeled potatoes, 20 g maltose, 10 g tryptone, 1.5 g MgSO_4_, 2.5 g KH_2_PO_4_, 20 mg thiamine (vitamin B1), and 1000 mL distilled water) was added in each well. A 5 μL aliquot of *F. pseudonygamai* supernatant solution was pipetted into the PM medium. Subsequently, 5 μL solution of blastospores (2 × 10^8^) were added to each well to achieve a final concentration of 1 × 10^7^ blastospores per mL, followed by mixing using a microporous quick shaker (Kylin-Bell, Haimen, China). For control wells extra pure ddH20 was added. The phenotype of blastospores were monitored daily under microscopy, and the data of transformation (%) of blastospores into pre-hyphae and hyphae was recorded after 4th and 8th day of inoculation. To accurately assess the overall increase in transformation (%) of blastospores following treatment with the *F. pseudonygamai* supernatant, the combined percentages of pre-hyphae and hyphae were quantified. Both the microscopic slide and hemocytometer were used to accurately observe and count transformation (%), respectively. For staining, Calcofluor White stain containing Evans Blue (Sigma Aldrich, Darmstadt, Germany) was used.

### 2.3. Transcriptomic Profiling of O. sinensis Treated with F. pseudonygamai Supernatant

For RNA-seq, blastospores were treated with fungal supernatant under the conditions described above (Section 2.2). Samples were centrifuged at 8000 rpm for 15 min at 10 °C and collected on the 4th and 8th days. Three biological replicates were used for each sample (control and treatment). Total RNA was extracted, and 12 cDNA libraries were prepared. Adapters were ligated, and Hieff NGS^®^ DNA Selection Beads were used for purification to select target fragments. PCR library amplification was performed, followed by sequencing on the Illumina Novaseq X Plus (Gene Denovo Biotechnology Co., Ltd., Guangzhou, China).

Raw reads obtained from sequencing (deposited in the NCBI SRA under BioProject accession PRJNA1344456) were processed using fastp (v0.18.0) to remove adapter-containing sequences, reads with >10% unknown nucleotides (N), and low-quality reads (>50% bases with Q-value ≤ 20). Bowtie2 (v2.2.8) was used to remove rRNA-mapped reads and the remaining clean reads were aligned to the *O. sinensis* reference genome (GenBank Assembly accession: GCA_052818365.1; BioProject: PRJNA1159368; Strain: PAD37595) using HISAT2 (v2.1.0). Transcript assembly was performed with StringTie (v1.3.1), and gene expression was quantified using RSEM (V1.3.0), with Transcripts Per Million (TPM) values calculated to measure transcript abundance. Differential expression analysis was conducted using DESeq2 for group comparisons and edgeR for pairwise sample comparisons, with differentially expressed genes (DEGs) identified based on absolute fold change and false discovery rate (FDR). Gene Ontology (GO) was used for functional annotation, and Kyoto Encyclopedia of Genes and Genomes (KEGG) pathway enrichment analysis identified significantly enriched pathways (FDR ≤ 0.05, *p* ≤ 0.05).

To validate RNA-sequencing results, 14 genes were selected based on DEGs and pathway analysis. The mRNA expression was analyzed by Quantitative real-time PCR (qRT-PCR). Total RNA (1 µg) was reverse transcribed into cDNA using TransScript^®^ One-Step gDNA Removal and cDNA Synthesis SuperMix (AT311, TransGen Biotech, Beijing, China), following the manufacturer’s instructions. For qRT-PCR, the reactions were performed on a Bio-Rad iQ2 optical system (Bio-Rad, Hercules, CA, USA) using 2X PerfectStart^®^ Green qPCR SuperMix (AQ-601, TransGen Biotech, Beijing, China). Furthermore, amplification program consisted of an initial denaturation at 95 °C for 2 min, followed by 40 cycles of 95 °C for 5 s and 60 °C for 10 s. A melting curve analysis (65–95 °C) was performed to confirm specificity. Several housekeeping genes were evaluated for normalization using the 2^−^^ΔΔCT^ method, and *TUBB* was identified as the most stable reference gene. Primer sequences are provided (Supplementary Table S1).

### 2.4. Assessment of Crude Extracts from F. pseudonygamai on O. sinensis Transformation

A total of 10 L of *F. pseudonygamai* was cultured for 3–4 days in PDB as described in Section 2.1. The culture was then vacuum-dried using a vacuum lyophilizer (model No. TF-SFD-0.5E; Shanghai Tianfeng Industrial Co., LTD., Shanghai, China), yielding approximately 63.4 g of dried powder. This powder was extracted three times with 1 L of 95% ethanol; each extraction was performed for 24 h. The extracted solution was filtered using a Buchner funnel and concentrated with a rotary evaporator at 40 °C. The resulting concentrate was further dried on a water bath to obtain the crude ethanol extract. The dried crude extract was fractionated using ethyl acetate and butanol. The organic solvents were concentrated, filtered with 0.22 μm filter and finally dried on water bath by working in laminar air flow cabinet. A total of 2.39 g of ethyl acetate extract and 8.2 g of butanol extract were obtained.

A bioassay was performed in a 96-well plate under the same conditions as detailed in Section 2.2, with the bacterial supernatant replaced by 10 μL crude extracts of ethyl acetate and butanol. Based on our pre-experimentation results on the effect of various DMSO dilutions (0.1%, 0.5%, 1%, 5%, and 10%) on blastospore transformation, 0.5% DMSO was selected as no significant difference was observed as compared to control. Consequently, the crude extract was dissolved in 0.5% DMSO and a range of concentrations from 500 µg/mL to 3.9 µg/mL were selected for bioassay. Additionally, 85 μL of PM medium and 5 μL solution of blastospores were added in each well. The data of transformation (%) was recorded after 4th day and 8th day of inoculation.

### 2.5. Isolation and Identification of Bioactive Compounds from F. pseudonygamai

For compound isolation, *F. pseudonygamai* was cultured in 30 L of PDB medium, ultimately yielding 321.7 g of dried powder using a vacuum drier, while PDB medium alone was processed in parallel as a control. After extraction with 95% ethanol, the crude ethanol extract was fractionated into 6.4 g of ethyl acetate extract and 17.2 g of butanol extract. Silica gel column chromatography was employed for compound isolation from both ethyl acetate and butanol crude extracts. Gradient elution was performed with various solvent systems, including petroleum ether-ethyl acetate solution (petroleum ether: acetate = 50:1, 25:1, 10:1, 5:1, 2:1, 1:1, 1:2, *v*/*v*), dichloromethane-methanol solution (dichloromethane: methanol = 50:1, 25:1, 10:1, 5:1, 2:1, 1:1, 1:2, *v*/*v*), and pure methanol.

Fractions were collected, concentrated, and combined based on similar polarity, as determined by thin layer chromatography (TLC). High-performance liquid chromatography (HPLC) (Agilent technologies G1316A with an InfinityLab Poroshell 120 EC-C18 column, 2.7 µm particle size, 4.6 × 100 mm, Santa Clara, CA, USA) was then used to analyze multiple peaks. Compounds exhibiting a single peak (>90% purity) in HPLC (Elite P3500 high-pressure constant-flow pump with a SinoChrom ODS-BP column, 10 µm particle size, 10.0 × 250 mm, Dalian, China) were subjected to ^1^*H*-NMR and ^13^*C*-NMR analysis. The spectra obtained from these analyses were carefully examined. Three pure compounds (adenosine, benzoic acid, and mannitol) were obtained. Adenosine and benzoic acid were dissolved in methanol, while mannitol was dissolved in distilled water.

### 2.6. Effect of Identified Compounds on O. sinensis Transformation

A bioassay was performed in a 96-well plate under the same conditions as detailed in Section 2.4, with the crude extract replaced by 10 μL of identified compounds. Adenosine (CAS 58-61-7), benzoic acid (CAS 65-85-0), and mannitol (CAS 69-65-8) were obtained from Shanghai Macklin Biochemical Technology Co., Ltd. (Shanghai, China) as ultra-pure grade powders for use in bioassays. The data of transformation (%) was recorded after 4th day and 8th day of inoculation.

### 2.7. Evaluation of Mannitol as a Key Metabolite Promoting O. sinensis Transformation

As mannitol from *F. pseudonygamai* was able to induce *O. sinensis* blastospore-to-hyphae transformation, two known inhibitors of mannitol biosynthesis enzymes were used, 3-nitrophenyl disulfide (CAS 537-91-7) and dihydrocelastrol (CAS 193957-88-9), to demonstrate mannitol function. These compounds were obtained as ultra-pure grade powders from Shanghai Macklin Biochemical Technology Co., Ltd., (Shanghai, China). These compounds target *mannitol dehydrogenase* (*MDH*) and *mannitol-1-phosphate dehydrogenase* (*M1PDH*). Each inhibitor was tested at concentrations ranging from 0.5 to 1000 µg/mL in liquid PDB medium to determine the maximum concentration that did not inhibit the growth of *F. pseudonygamai*. Cultures were incubated at described above (Section 2.1), and fungal growth was assessed by measuring optical density at 600 nm (OD_600_) after 4 days. The highest concentration showing no significant growth inhibition was selected for further use in bioassays. Sodium chloride (NaCl) was evaluated as a potential agonist of *M1PDH*.

For the transformation bioassay, 96-well microtiter plates were prepared as described in Section 2.2. Each well contained 90 µL PM medium, 5 µL of *F. pseudonygamai* supernatant (pretreated with the inhibitor, the agonist or a combination of both), and 5 of µL *O. sinensis* blastospore suspension. Transformation assays were conducted under five conditions: (i) positive control (CK^+^) with standard PM medium (PBS was added); (ii) negative control (CK^−^) with spores treated with inhibitors or agonist alone; (iii) fungal supernatant alone; (iv) fungal supernatant from *F. pseudonygamai* cultures treated with inhibitor or agonist; and (v) fungal supernatant from cultures treated with both inhibitor and agonist. Plates were incubated at 16 °C, and blastospore-to-hyphae transformation was recorded on days 4 and 8 post-inoculation.

### 2.8. Measurement of Mannitol Concentration from F. pseudonygamai Supernatant by Refractive Index Detection High-Performance Liquid Chromatography (RID-HPLC)

To confirm the involvement of mannitol in enhancing *O. sinensis* transformation efficiency, mannitol levels were quantified in *F. pseudonygamai* supernatant treated with a biosynthesis inhibitor, agonist and with inhibitor plus agonist (rescue experiment).

An Agilent 1290 HPLC system equipped with a Phenomenex Luna NH_2_ column (5 µm, 100 Å, 4.6 × 250 mm, Santa Clara, CA, USA) and a refractive index detector (RID) was used for analysis. Isocratic elution was performed with a mobile phase consisting of 35% water (solvent A) and 65% acetonitrile (solvent B). The flow rate was set to 1.0 mL/min, and the injection volume was 20 µL. The column and RID detector temperatures were maintained at 35 °C. A standard curve was prepared using serial dilutions of analytical-grade mannitol dissolved in distilled water. Mannitol concentration in each sample was quantified by comparing the RID peak area to the standard curve generated from known mannitol concentrations.

### 2.9. Gene Expression Analysis (MDH and M1PDH) by qRT-PCR

To further confirm the involvement of mannitol biosynthesis in promoting *O. sinensis* transformation, the expression levels of both *MDH* and *M1PDH* were evaluated in *F. pseudonygamai* treated with 3-nitrophenyl disulfide (5 µg/mL) alone or in combination with NaCl (5 mg/mL) as a rescue experiment. For RNA extraction, 40 mL cultures of *F. pseudonygamai* were treated and cultured under the same conditions as described above (Section 2.1). Cultures were centrifuged at 8000 rpm for 10 min at 4 °C, and the resulting mycelial pellets were collected. These were immediately flash-frozen in liquid nitrogen and ground to a fine powder using a pre-chilled mortar and pestle. Approximately 500–1000 mg of powdered pellets were used for total RNA isolation using the HiPure Universal RNA Mini Kit (Magen Biotechnology Co., Ltd., Guangzhou, China), following the manufacturer’s instructions.

Total RNA (1 µg per sample) was then reverse transcribed into complementary DNA (cDNA) and qRT-PCR were performed using a Bio-Rad iQ2 real-time PCR system as described above (Section 2.3).

### 2.10. Statistical Analysis

Data collection and statistical analysis were performed using GraphPad Prism 9.0 and Microsoft Excel. Data are presented as mean ± standard deviation (SD). The choice of statistical test depended on the experimental design. For comparisons among more than two groups, a one-way analysis of variance (ANOVA) was performed. For comparisons between two groups, an unpaired two-tailed Student’s *t*-test was used. *p*-values, with * *p* < 0.05; ** *p* < 0.01; *** *p* <0.001 denoting statistically significant differences, while ns means not significantly different at *p* > 0.05. Error bars representing standard deviation (SD). The specific statistical test applied to each dataset is stated in the corresponding figure legend.

## 3. Results

### 3.1. Impact of F. pseudonygamai Supernatant on O. sinensis Transformation

**Figure 1:**
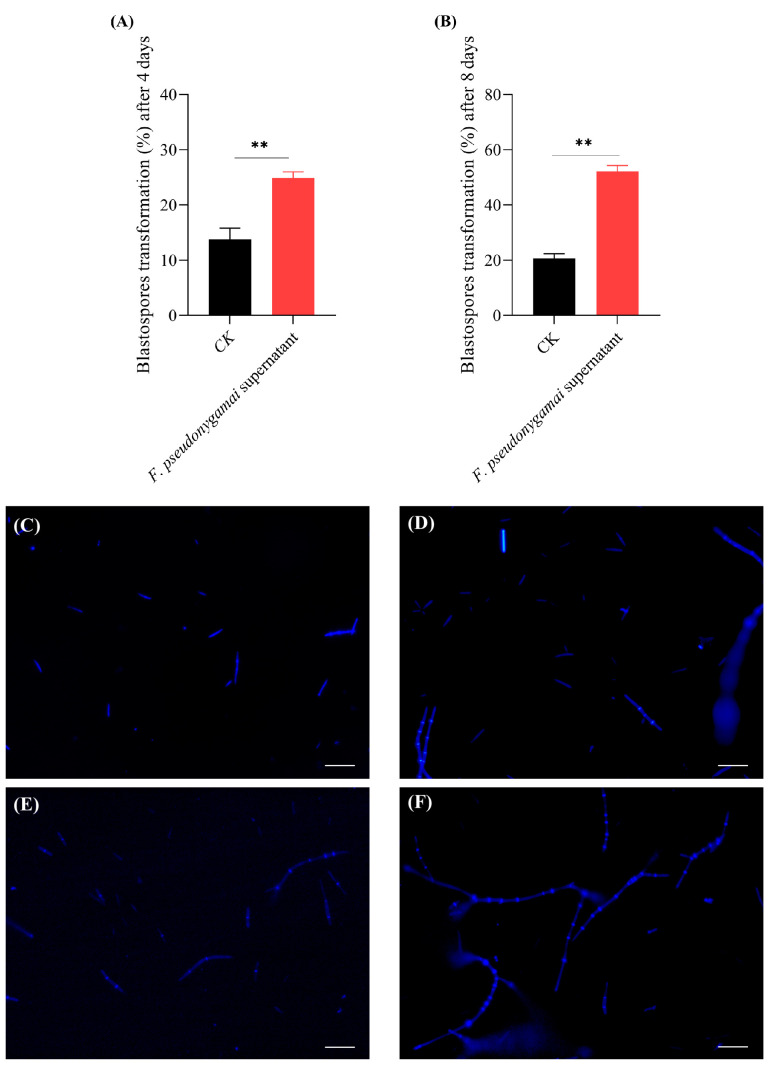
Percentage transformation of blastospores into pre-hyphae and hyphae following treatment with *F. pseudonygamai* supernatant. (**A**) Blastospores transformation (%) after 4 days. (**B**) Blastospores transformation (%) after 8 days. (**C**) Hemocytometer-based observation of transformation from the control (CK) group at day 4. (**D**) Hemocytometer-based observation of transformation from the CK group at day 8. (**E**) Hemocytometer-based observation of transformation in the treatment group at day 4. (**F**) Hemocytometer-based observation of transformation in the treatment group at day 8. Scale bar 50 μm. Data are presented as mean ± SD and analyzed using a two-tailed unpaired Student’s *t*-test (** *p* < 0.01).

After 4 days, blastospores of *O. sinensis* treated with *F. pseudonygamai* supernatant showed a transformation rate of 24.9% in the treatment group, while the control group displayed transformation rate of 13.7% (Figure 1A). The transformation rate was 52.2% in the treatment group compared to 20.6% in the control group, indicating a significant increase of 31.6% after 8 days of treatment with *F. pseudonygamai* supernatant (Figure 1B).

The results indicated that supernatant of *F. pseudonygamai* can significantly increase the transformation of blastospores into pre-hyphae and hyphae, ultimately affecting the infection rate of *O. sinensis*. To further investigate the molecular mechanisms underlying this increased transformation, transcriptomic analysis was performed on *O. sinensis* following treatment with *F. pseudonygamai* supernatant.

### 3.2. Transcriptomic Profiling of O. sinensis in Response to F. pseudonygamai Supernatant

Next-generation Illumina RNA sequencing was performed for 12 libraries, including both control (CK4 and CK8) and treatment groups (T4 and T8). High-quality clean reads were obtained and mapped to the *O. sinensis* reference genome (GenBank Assembly: GCA_052818365.1), with a mapping rate > 92% across all the libraries. Gene expression levels were quantified using TPM (Transcripts Per Million) and DEGs were identified. Functional enrichment analysis mapped DEGs to GO terms (http://www.geneontology.org/) and KEGG pathways, revealing significant associations with various biological processes and metabolic/signaling pathways.

**Figure 2:**
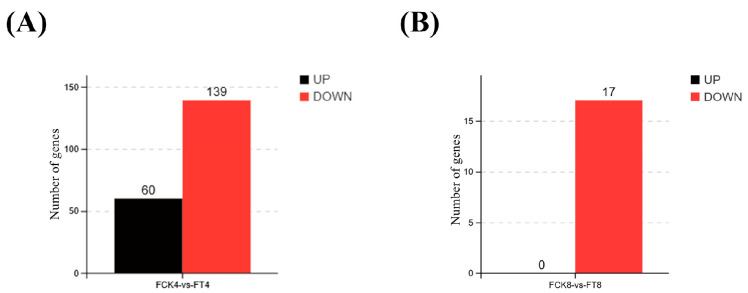
Differentially Expressed Genes (DEGs) in *Ophiocordyceps sinensis* blastospores in response to *Fusarium pseudonygamai* supernatant. (**A**,**B**) Bar graphs show the number of significantly upregulated (UP) and downregulated (DOWN) genes in the treatment group compared to the control at (**A**) day 4 and (**B**) day 8.

After 4 days of treatment, a total of 60 DEGs were upregulated and 139 DEGs were downregulated between CK4 (control group at day 4) and T4 (treatment group at day 4) (Figure 2A). By day 8, no genes were significantly upregulated, and only 17 DEGs were downregulated (Figure 2B). Key genes involved in oxidative phosphorylation (*CYC1*, 2.0-fold), nitric oxide detoxification (*hmp*, 2.7-fold), redox homeostasis (*gedE*, 2.2-fold), and amino acid metabolism (*fahA*, 2.3-fold) were significantly upregulated on day 4. These genes enhance mitochondrial function, alleviate nitrosative stress, maintain intracellular redox balance, and supply metabolic intermediates that support the energy requirements and cellular remodeling necessary for hyphal development. The upregulation of these genes on day 4 indicates that transcriptional reprogramming associated with hyphal development was initiated early. This aligns with the bioassay results, where the most pronounced spore-to-hypha transition was observed by day 8, suggesting that the molecular signaling driving morphological change was transcriptionally established by day 4 and had largely progressed by day 8, as reflected by the reduced number of DEGs. Complete lists of all DEGs and associated GO terms are provided in Supplementary File (Supplementary Files S1–S4).

**Figure 3:**
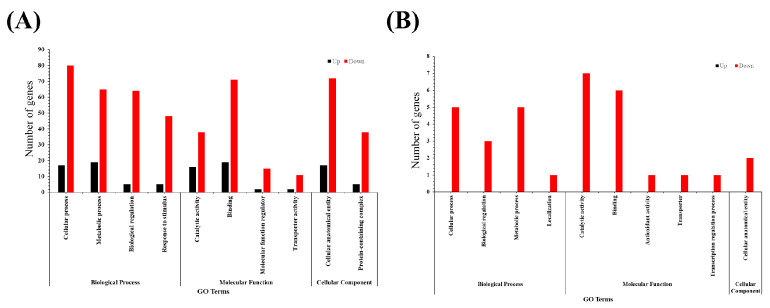
Gene Ontology (GO) enrichment of *Ophiocordyceps sinensis* genes following *Fusarium pseudonygamai* supernatant treatment. (**A**,**B**) Significantly enriched terms at (**A**) day 4 and (**B**) day 8.

GO and KEGG enrichment analyses were performed to gain insight into the molecular changes during spore-to-hypha transformation. On day 4, GO enrichment highlighted key categories such as cellular processes and catalytic activity, consistent with the early activation of metabolic and cellular functions (Figure 3A). These early transcriptional shifts likely support energy mobilization and signaling adjustments conducive to transformation. Meanwhile, KEGG analysis revealed significant upregulation of oxidative phosphorylation and tyrosine metabolism (FDR < 0.05), suggesting enhanced mitochondrial activity and ATP production essential for early developmental transitions (Figure S1A). By day 8, GO enrichment analysis showed broader downregulation of genes related to basic cellular and metabolic functions (Figure 3B), suggesting a strategic reallocation of resources to support sustained hyphal growth. Additionally, KEGG pathway analysis showed widespread upregulation of biosynthetic, metabolic, and differentiation-related pathways, indicating that major transcriptional changes were largely completed (Figure S1B).

#### Validation of RNA-Seq

To validate the RNA-seq results, the mRNA expression of *CYC1*, *hmp*, *gedE*, *fahA*, *FDB3*, *nuo-21*, and *rad18* was evaluated on day 4, while mRNA expression of *Chdh*, *FLC1*, *het-6*, *IP5P9*, *pkc1*, *pik1*, and *klp5* was evaluated on day 8 using qRT-PCR (Figure S2). On day 4, *CYC1*, *hmp*, *gedE* and *fahA* were upregulated with relative expression of 2.4, 2.6, 2.3 and 1.7, respectively, compared to the control, supporting their role in promoting the blastospore-to-hyphae transformation (Figure S2A). By day 8, all selected genes showed downregulation (Figure S2B). These results were consistent with the RNA-seq data, confirming the reliability of the gene expression profiles.

### 3.3. Bioactivity of Crude Extracts from F. pseudonygamai

**Figure 4:**
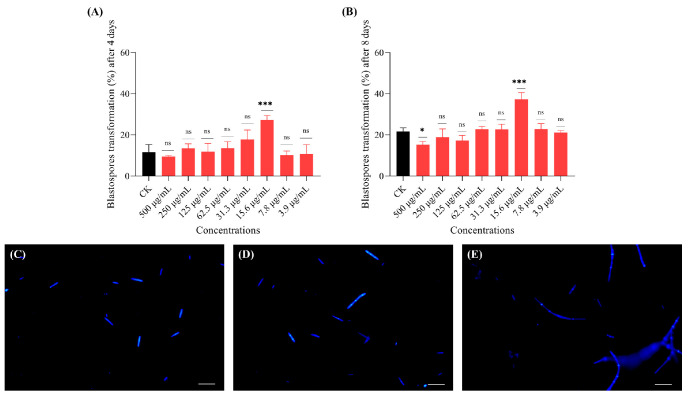
Effect of crude butanol extract from *Fusarium pseudonygamai* on *Ophiocordyceps sinensis* blastospore transformation. (**A**,**B**) Bar graphs showing the effect of butanol crude extract on *O. sinensis* blastospore transformation at (**A**) day 4 and (**B**) day 8. (**C**) Confocal microscopic image from control group (CK) at day 8. (**D**,**E**) Confocal microscopic image of blastospores treated with 15.6 µg/mL butanol extract at day 4 and day 8, respectively. Scale bar 50 μm. Data are presented as mean ± SD and analyzed using one-way ANOVA followed by Dunnett’s post hoc test (* *p* < 0.05, *** *p* < 0.001, ns = not significant).

To further investigate the effect of *F. pseudonygamai* supernatant on the transformation of *O. sinensis* blastospores, an experiment was conducted using crude ethyl acetate and butanol extracts from the fungal isolate. Among the 8 selected concentrations of butanol extract, our results indicated a significant increase in the transformation (around 15%) at 15.6 µg/mL after 4 days and 8 days of inoculation, respectively (Figure 4). At day 4 a higher proportion of pre-hyphal structures were observed (Figure 4D), while by day 8 more extensive hyphal development had occurred (Figure 4E), indicating progressive morphological transformation over time. Furthermore, at higher concentration (500 µg/mL), the butanol crude extract significantly reduced the transformation after 8 days. However, no significant increase in the transformation (%) was observed following treatment with the selected concentrations of ethyl acetate crude extract (Figure S3).

Assessing the bioactivity of the crude extract revealed its potential role in promoting transformation, prompting further compound isolation and identification to determine the active constituents.

### 3.4. Isolation and Identification of Bioactive Compounds

**Figure 5:**
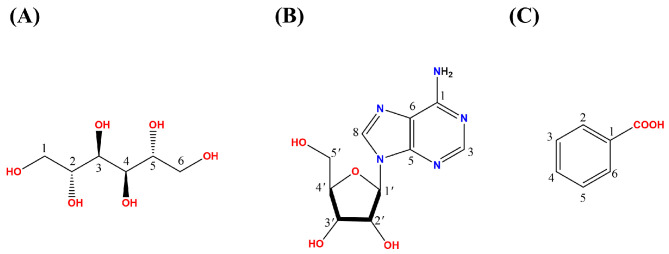
Structures of three pure compounds isolated from the butanol crude extract of *Fusarium pseudonygamai*: (**A**) Mannitol (FB30), (**B**) Adenosine (FB25-27), and (**C**) Benzoic acid (FB30-38gi11-12).

From our butanol crude extract, three pure compounds, namely adenosine (FB25-27, 4 mg) benzoic acid (FB30-38gi11-12, 6 mg) and mannitol (FB30, 10 mg), were isolated and identified (Figure 5), using 1D NMR spectroscopic analysis (Figure S4) and literature comparisons [20,21,22]. The spectroscopic data are as follows:

Adenosine (FB25-27): White powder; ^1^H-NMR (600 MHz, MeOD-*d*_4_) δH (ppm): 8.40 (1H, s, H-3), 8.35 (1H, s, H-8), 5.90 (1H, d, *J =* *6.5* Hz, H-1′), 4.98 (1H, d, *J = 4.8* Hz, H-2′), 4.44 (2H, br s, 1-NH_2_), 4.34 (1H, m, H-3′), 4.17 (1H, q, *J = 3.0* Hz, H-4′), 3.90 (1H, dd, *J = 2.4, 12.6* Hz, H-5′a), 3.75 (1H, dd, *J = 3.0, 12.6* Hz, H-5′b); ^13^C-NMR (150 MHz, MeOD-*d*_4_) δC (ppm): 154.6 (C-1), 152.1 (C-3), 150.1 (C-5), 140.6 (C-8), 121.2 (C-6), 91.3 (C-1′), 89.8 (C-4′), 74.0 (C-2′), 71.2 (C-3′), 62.0 (C-5′).

Mannitol (FB30): White powder, ^1^HNMR (600 MHz, DMSO-*_d_*_6_) δH (ppm): 4.48 (2H, d, *J = 5.48* Hz, H-3,4), 4.12 (2H, t, *J = 5.69* Hz, H-2,5), 4.11 (2H, d, *J = 7.05* Hz, H-1), 3.60–3.57 (2H,m, H-8), 3.51 (2H, m, H-5b), 3.49–3.43 (2H, m, H-6), 340–3.34 (2H, m, H-7); ^13^CNMR (150 MHz, DMSO-*_d_*_6_) δC (ppm): 71.7 (C-2,5), 70.1 (C-3,4), 64.3 (C-1,6).

Benzoic acid (FB30-38gi11-12): White powder; ^1^H-NMR (600 MHz, MeOD-*d*_4_) δH (ppm): 7.80 (5H, m, H-2,4,3,5,6); ^13^C-NMR (150 MHz, MeOD-*d*_4_) δC (ppm): 174.80 (C-7), 135.9 (C-4), 129.0 (C-1), 128.5 (C-2,6), and 126.9 (C-3,5).

### 3.5. Mannitol Is the Key Metabolite from F. pseudonygamai Promoting Blastospore Transformation

**Figure 6:**
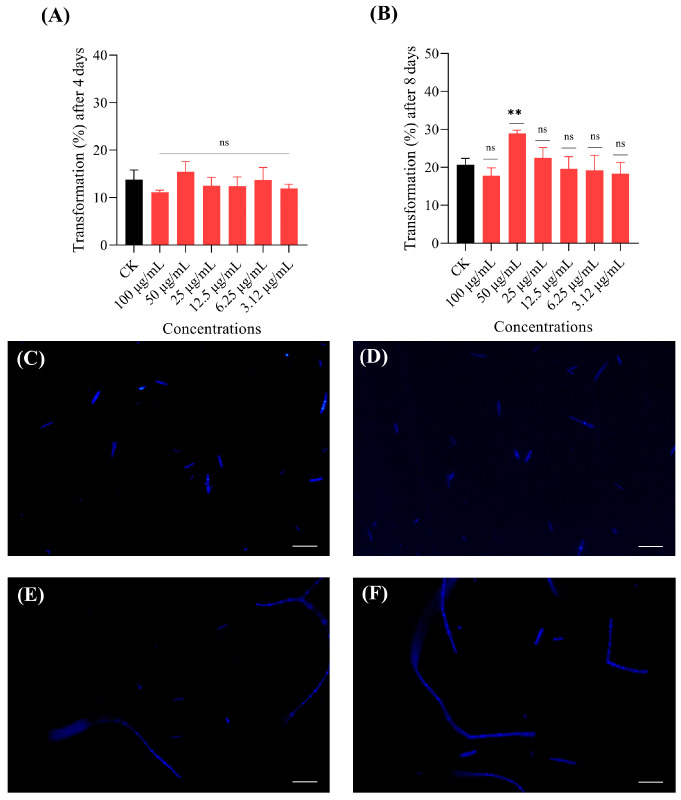
Effects of mannitol on *O. sinensis* blastospore transformation. (**A**,**B**) Bar graphs showing the effect of mannitol on blastospore transformation at (**A**) day 4 and (**B**) day 8. (**C**,**D**) Confocal microscopic images from control (CK) group at (**C**) day 4 and (**D**) day 8. (**E**,**F**) Confocal microscopic images from mannitol treated group (50 µg/mL) at (**E**) day 4 and (**F**) day 8. Scale bar 50 μm. Data are presented as mean ± SD and analyzed using one-way ANOVA followed by Dunnett’s post hoc test (** *p* < 0.01, ns = not significant).

A significant increase in transformation rate (8.3%) was observed after 8 days of treatment with mannitol at a concentration of 50 µg/mL (Figure 6). Among all the selected concentrations, adenosine had no significant effect on the transformation (%) of *O. sinensis* spores after 4th and 8th days of treatment (Figure S5A,B). Likewise, benzoic acid did not influence the transformation rate after 4 and 8 days of treatment (Figure S5C,D). These results suggest that mannitol plays a key role in promoting blastospore transformation into pre-hyphae and hyphae.

### 3.6. Effect of Mannitol Biosynthesis Modulators on O. sinensis Transformation

Prior to assessing their impact on *O. sinensis* transformation, we first determined whether the mannitol biosynthesis modulators affected the growth of *F. pseudonygamai*, to ensure that any observed effects on transformation were not due to impaired fungal viability. Our results showed that dihydrocelastrol had no inhibitory effect on *F. pseudonygamai* growth at any tested concentration, whereas 3-nitrophenyl disulfide significantly inhibited growth at concentrations ≥ 10 µg/mL, with no effect observed at 5 µg/mL (Figure S6). Based on these results, 3-nitrophenyl disulfide (5 µg/mL) and dihydrocelastrol (50 µg/mL and 100 µg/mL) were selected for subsequent transformation assays.

**Figure 7:**
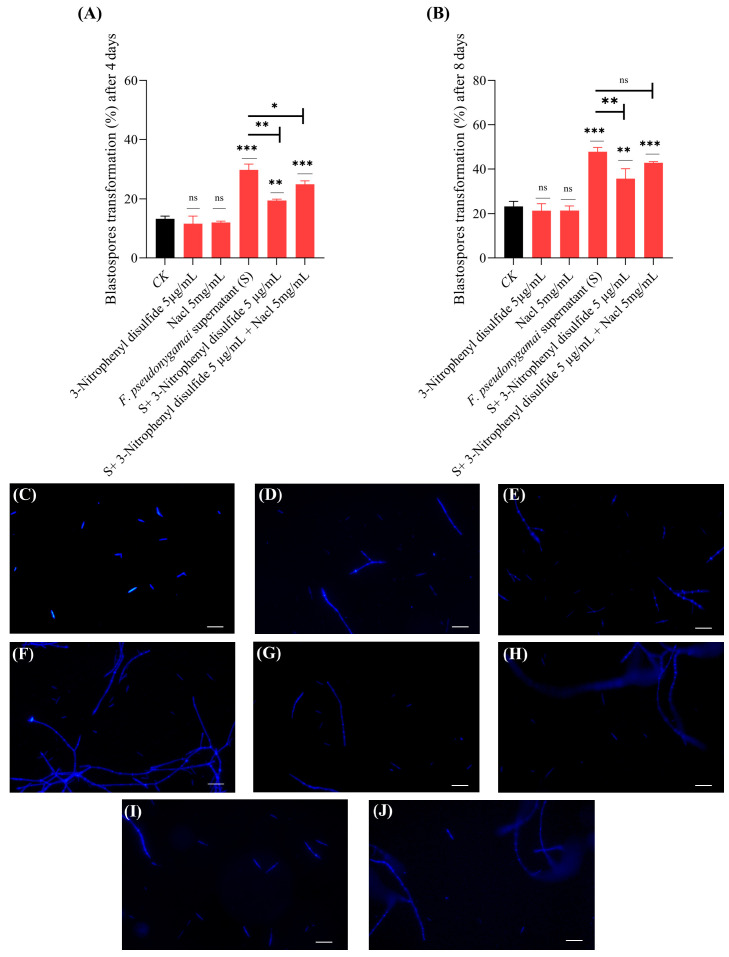
Effect of *F. pseudonygamai* supernatant from cultures treated with mannitol modulators on *O. sinensis* blastospores transformation (%). (**A**,**B**) Transformation rates at Day 4 (**A**) and Day 8 (**B**) under different treatments. (**C**,**D**) Microscopic images of fungal structures in the control group at Day 4 (**C**) and Day 8 (**D**). (**E**,**F**) Fungal structures treated with *F. pseudonygamai* supernatant alone at Day 4 (**E**) and Day 8 (**F**), showing enhanced transformation. (**G**,**H**) Blastospores of *O. sinensis* incubated with *F. pseudonygamai* supernatant collected from cultures grown with 3-nitrophenyl disulfide at Day 4 (**G**) and Day 8 (**H**). (**I**,**J**) Blastospores of *O. sinensis* treated with both inhibitor and agonist (NaCl) at Day 4 (**I**) and Day 8 (**J**). Scale bar 50 μm. Data are presented as mean ± SD and analyzed using one-way ANOVA followed by Dunnett’s post hoc test (* *p* < 0.05, ** *p* < 0.01, *** *p* < 0.001, ns = not significant).

Results indicated that 3-nitrophenyl disulfide (negative control) had no direct impact on transformation rate of *O. sinensis* blastospores. Interestingly, treatment with fungal supernatant significantly enhanced spore transformation, increasing from 13% in the control to 29% on Day 4 and from 23% to 47% on Day 8 (Figure 7). Treatment of supernatant derived from 3-nitrophenyl disulfide–treated *F. pseudonygamai* cultures reduced transformation rate, resulting in 19% on Day 4 and 35% on Day 8, compared with untreated supernatant (Figure 7A,B). These results suggest that 3-nitrophenyl disulfide might inhibited the production of transformation-enhancing metabolites, such as mannitol. Microscopic images show fungal structures including blastospores, pre-hyphae and hyphae under various conditions (Figure 7C–J). Supernatant from NaCl-treated cultures did not increase transformation compared to untreated supernatant, indicating that NaCl elevated mannitol levels beyond the optimal range, thereby limiting its ability to promote transformation.

Interestingly, the inhibitory effect of 3-nitrophenyl disulfide was reversed by co-treatment with NaCl, improving transformation rates to 24% on Day 4 and 42% on Day 8. By Day 8, the transformation rate in the NaCl rescue group (42%) was significantly higher than in the inhibitor-treated group (35%) and comparable to that observed with the untreated supernatant (47%) (Figure 7A,B). These results indicated that NaCl (agonist) effectively counteracted the suppression caused by 3-nitrophenyl disulfide (inhibitor), likely by restoring mannitol synthesis via *M1PDH* activation.

**Figure 8:**
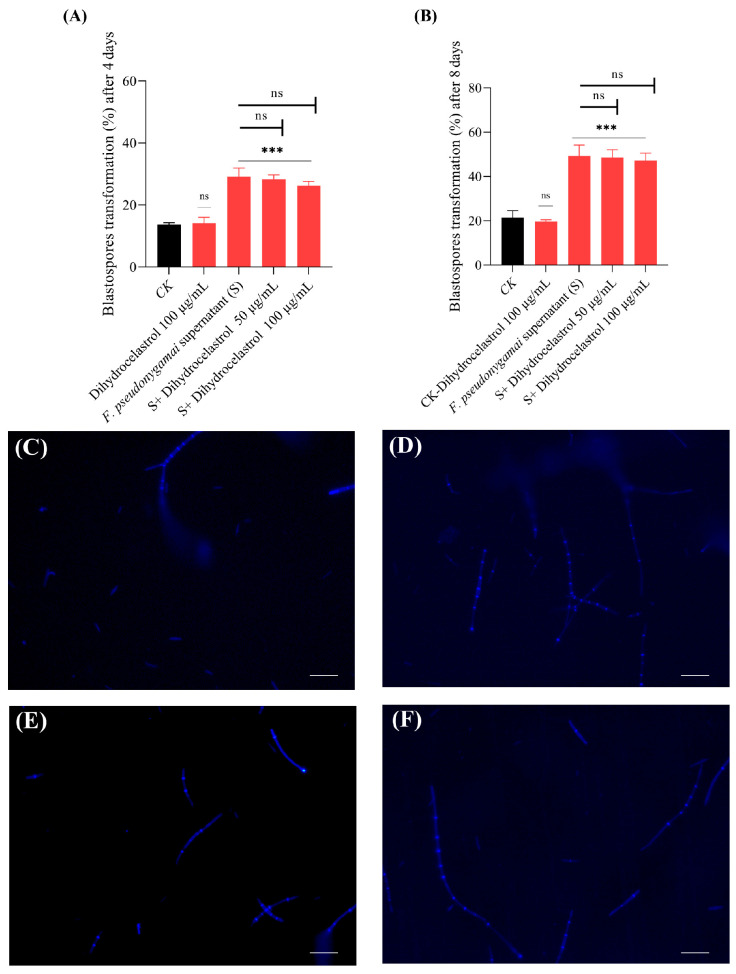
Effect of dihydrocelastrol-treated *F. pseudonygamai* supernatants on blastospores transformation of *O. sinensis*. (**A**,**B**) Transformation rates (%) at Day 4 (**A**) and Day 8 (**B**) for control (CK) and dihydrocelastrol-treated supernatant groups, respectively. (**C**–**F**) Confocal microscopic images of *O. sinensis* fungal structures: (**C**) CK at Day 8 (**C**); (**D**) treated with *F. pseudonygamai* supernatant at Day 8; (**E**) treated with dihydrocelastrol-treated *F. pseudonygamai* supernatant at Day 4; (**F**) treated with dihydrocelastrol-treated *F. pseudonygamai* supernatant at Day 8. Scale bar 50 μm. Data are presented as mean ± SD and analyzed using one-way ANOVA followed by Dunnett’s post hoc test (*** *p* < 0.001, ns = not significant).

In contrast, supernatants obtained from dihydrocelastrol-treated cultures (at both 50 and 100 µg/mL) did not significantly affect transformation of *O. sinensis* compared to untreated supernatant. Transformation rates remained between 47% and 49%, suggesting that dihydrocelastrol did not effectively suppress the biosynthesis of transformation-promoting metabolites under the tested conditions (Figure 8).

Our results demonstrate that 3-nitrophenyl disulfide impairs transformation of *O. sinensis* spores, likely through interference with metabolite production such as mannitol. This effect can be counteracted by NaCl, supporting its potential role in restoring mannitol level involved in transformation, possibly by enhancing *M1PDH* activity as an agonist. Collectively, our results suggest that mannitol’s effect is concentration-dependent, with only specific levels enhancing transformation.

### 3.7. Quantification of Mannitol Concentration in F. pseudonygamai Supernatants by RID-HPLC

**Figure 9:**
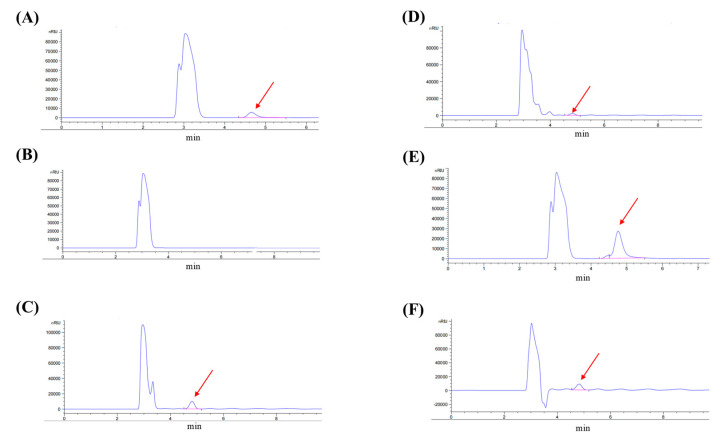
RID-HPLC chromatograms of mannitol quantification in *F. pseudonygamai* culture supernatants under different treatment conditions. (**A**) Mannitol standard showing retention time at 4.7 min. (**B**) Blank control (no peak). (**C**) Untreated culture supernatant. (**D**) Supernatant from culture treated with 3-Nitrophenyl disulfide. (**E**) Supernatant from culture treated with NaCl. (**F**) Co-treatment with NaCl and 3-Nitrophenyl disulfide.

Our results indicated that the chromatographic peak for mannitol was observed at approximately 4.7 min in the standard and sample runs (Figure 9). This identity of the mannitol peak in the sample was confirmed by co-elution with the standard (0.5 mg/mL in distilled water) (Figure 9A). As a blank control, no detectable peak was observed at this retention time (Figure 9B). In the control group (CK), mannitol concentration was 0.8 mg/mL (Figure 9C). Given that 5 µL of supernatant was added to 100 µL of blastospore culture in the bioassay (Section 3.1), the effective mannitol concentration was approximately 40 µg/mL. Such concentration aligns with our results with pure mannitol treatment (Section 3.5), where 50 µg/mL of pure mannitol significantly enhanced transformation efficiency, reinforcing the concentration-dependent role of mannitol.

Culturing *F. pseudonygamai* with 3-nitrophenyl disulfide at 5 µg/mL significantly reduced mannitol production to 0.18 mg/mL (Figure 9D). In contrast, the addition of NaCl in the culture media markedly increased mannitol production to 2.6 mg/mL (Figure 9E). Notably, co-treatment of 3-nitrophenyl disulfide with NaCl restored mannitol production to 0.79 mg/mL (Figure 9F), comparable to the untreated control. Both 3-nitrophenyl disulfide (an *M1PDH* inhibitor) and NaCl (an *M1PDH* agonist) significantly affected mannitol production, with NaCl enhancing and 3-nitrophenyl disulfide suppressing *M1PDH* levels, thereby directly impacting mannitol biosynthesis and transformation efficiency. Overall, the RID-HPLC results reinforce the concentration-dependent role of mannitol in enhancing *O. sinensis* blastospore transformation.

Furthermore, we also detected a very low concentration of mannitol (2.9 µg/mL) in the supernatant of *O. sinensis* culture media after 25 days of inoculation by HPLC-MS. This substantial difference, along with the potent stimulation of transformation by exogenous mannitol from *F. pseudonygamai* supernatant, suggests that the microbiota-derived mannitol acts as a signaling molecule to enhance the blastospore transformation in *O. sinensis*.

### 3.8. Gene Expression Analysis of MDH and M1PDH in Response to Mannitol Modulator Treatment

**Figure 10:**
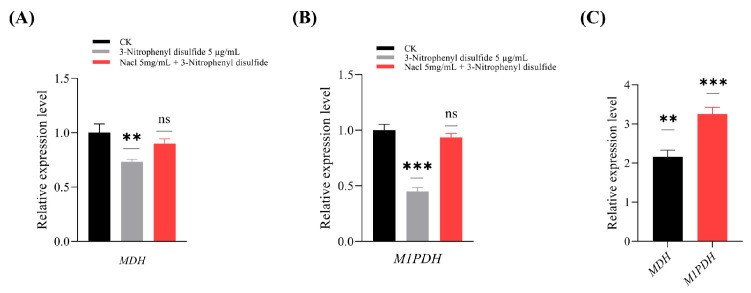
Relative expression level of mannitol biosynthesis genes in *F. pseudonygamai* under different modulator treatments. (**A**) Relative expression of *MDH*. (**B**) Relative expression of *M1PDH*. (**C**) Relative expression level of mannitol biosynthesis genes under control condition compared to *TUBB*. Expression levels are shown for control, inhibitor (3-Nitrophenyl disulfide), and inhibitor + agonist (NaCl) treatments. Data are presented as mean ± SD and analyzed using one-way ANOVA followed by Dunnett’s post hoc test (** *p* < 0.01, *** *p* < 0.001, ns = not significant).

To further investigate the role of mannitol biosynthesis in enhancing *O. sinensis* transformation, the expression of two key mannitol-related genes (*M1PDH* and *MDH*) was analyzed in *F. pseudonygamai* under modulator treatments after 4 days of culture (Figure 10). Treatment with the inhibitor 3-nitrophenyl disulfide (5 µg/mL) significantly downregulated both genes, reducing *M1PDH* expression to 0.45- and *MDH* to 0.73-fold relative to the control. Co-treatment with NaCl (5 mg/mL) restored expression, with *M1PDH* and *MDH* reaching 0.93- and 0.89-fold of the control level, respectively (Figure 10A,B). The more pronounced suppression of *M1PDH* compared to *MDH* suggests that 3-nitrophenyl disulfide primarily targets *M1PDH*, and that NaCl effectively reverse this inhibitory effect. These results indicate that mannitol levels in the supernatant were closely linked to the expression of *M1PDH* and *MDH*, with modulator treatments (3-nitrophenyl disulfide and NaCl) significantly altering gene expression and consequently affecting mannitol production.

Additionally, *MDH* and *M1PDH* showed expression levels of 2.1- and 3.2-fold, respectively, compared to the reference gene *TUBB* under control conditions, indicating active mannitol biosynthesis in *F. pseudonygamai* (Figure 10C).

## 4. Discussion

In our previous study, high-throughput sequencing of 16S rRNA and ITS regions revealed diverse and distinct microbial communities inhabiting the gut and hemolymph of *T. xiaojinensis*, with notable shifts observed upon *O. sinensis* infection [15]. These findings suggested that host-associated microbiota may influence fungal development and infection dynamics. Based on these insights, over 100 microbial isolates were screened for their ability to induce blastospore-to-hyphae transformation in *O. sinensis*. Among these, *F. pseudonygamai* significantly promoted hyphal development. To elucidate the mechanisms underlying these effects, the present study focused on characterizing the interaction between *F. pseudonygamai* and *O. sinensis*, shedding light on the microbial contributions to fungal morphogenesis and development. Our results indicated that treatment with *F. pseudonygamai* supernatant led to a time-dependent enhancement of transformation, with an 11.2% increase observed by day 4 and a marked 31.6% increase by day 8. These findings align with previous reports suggesting that both external environmental microbiota and internal host microbes play critical roles in regulating *O. sinensis* infection and development [14,15,23].

Transcriptomic sequencing is a powerful tool for uncovering the molecular mechanisms underlying complex biological transitions by providing a comprehensive view of gene expression dynamics [24,25]. In this study, transcriptomic analysis revealed distinct stage-specific gene expression changes during *O. sinensis* transformation induced by *F. pseudonygamai* supernatant. On day 4, 60 genes were upregulated and 139 downregulated, indicating early transcriptional reprogramming. Notably, genes such as *CYC1*, *hmp*, *gedE*, and *fahA* were significantly upregulated, supporting energy production, stress response, and metabolic activation for hyphal growth. These genes have previously been implicated in fungal development, and their expression patterns in this study are consistent with known roles in morphogenesis. For example, *CYC1* encodes cytochrome c which is a central component of the mitochondrial electron transport chain that drives ATP production through oxidative phosphorylation. In *Podospora anserina*, disruption of *CYC1* impairs mitochondrial function, leading to slow growth, poor germination, and underdeveloped mycelium, emphasizing its role in energy-dependent hyphal elongation [26,27]. *hmp*, which encodes flavohemoglobin, exhibits developmentally regulated expression with peak levels during conidial germination. This regulation plays a critical role in mitigating nitrosative stress by detoxifying nitric oxide, thereby promoting early fungal development and facilitating both spore germination and hyphal elongation, as demonstrated in *Botrytis cinerea* [28]. Similarly, in fungi, glutathione metabolism has been closely tied to morphogenesis. In *Candida albicans* and *Aureobasidium pullulans*, depletion of intracellular glutathione (GSH) has been associated with the yeast-to-hyphae transition, suggesting that low GSH levels may facilitate morphological switching [29,30,31]. However, in *Histoplasma capsulatum*, overexpression of GSH biosynthesis genes (*gsh1* and *gsh2*) maintained cells in the yeast form, further supporting the role of GSH in regulating fungal morphology. Similarly, *Penicillium chrysogenum* exhibited yeast-like growth under high GSH conditions. However, later studies in *C. albicans* proposed that GSH depletion may result from, rather than initiate, filamentation, indicating a complex regulatory relationship between GSH levels and hyphal development [32,33]. *fahA*, involved in tyrosine catabolism, supplies carbon intermediates to the TCA cycle for energy production and stress resistance [34]. The coordinated upregulation of these genes in *O. sinensis* suggests an integrated metabolic and stress response essential for blastospore-to-hyphae transformation. GO and KEGG enrichment analyses further confirmed upregulation of oxidative phosphorylation, tyrosine metabolism, and proteasome pathways, consistent with increased biosynthetic and cellular activity. By day 8, transcriptional changes stabilized, with no upregulated genes and only 17 downregulated, suggesting early initiation and completion of the transformation program. These results align with phenotypic observations and highlight the role of microbial cues in priming *O. sinensis* for developmental transition.

Our results with the bioassays with the crude butanol extract from *F. pseudonygamai* culture showed a significant 15% increase in transformation at 15.6 µg/mL after both 4 and 8 days of treatment, compared to the control. Transformation progressed from pre-hyphae at day 4 to well-developed hyphae by day 8. Subsequently, among the three isolated compounds, only mannitol significantly enhanced blastospore-to-hyphae transformation emerging as a key effector. Functional validation using biosynthesis modulators further confirmed its role. Inhibition of mannitol production by 3-nitrophenyl disulfide reduced transformation efficiency, and co-treatment with NaCl effectively restored both mannitol levels and transformation rates. These effects were supported by RID-HPLC quantification and qRT-PCR analysis, which revealed that changes in *M1PDH* and *MDH* expression correlated with mannitol levels in the culture supernatant. Notably, the transformation-promoting effect was concentration-dependent, with optimal results around 40–50 µg/mL in vitro. Likewise, recent in vivo studies demonstrated that mannitol accumulates specifically during the mummification phase of *T. xiaojinensis* infected by *O. sinensis*, coinciding with fungal dimorphism [16]. Direct injection of mannitol into the hemolymph of infected larvae significantly accelerated mummification rates, suggesting its active role in mediating fungal pathogenicity and host manipulation [16,35], although the hemolymph full of *O. sinensis* blastospores or prehyphae also contains other microorganisms [15]. Whether exogenous mannitol directly induces the larval mummification rates or stimulates other microorganisms to induce the larval mummification rates needs to be confirmed. Anyway, our findings demonstrate the direct induction of dimorphic transition in pure *O. sinensis* fungus, by in vitro observations, reinforcing mannitol’s dual function as a morphogenetic signal in the *O. sinensis* life cycle. Further experiments should explore whether other metabolites from *F. pseudonygamai* have similar or stronger effects on *O. sinensis* transformation.

## 5. Conclusions

This study demonstrates that microbial metabolites, particularly mannitol isolated and produced by *F. pseudonygamai*, a fungus associated with *T. xiaojinensis* larvae, play a crucial role in promoting the transformation of *O. sinensis* blastospores to hyphae. Treatment with *F. pseudonygamai* supernatant significantly enhanced transformation, which was accompanied by transcriptional activation of genes associated with energy metabolism, stress response, detoxification, and hyphal development. Functional validation through modulation of mannitol biosynthesis further confirmed its regulatory role. These findings provide new insights into microbiota–fungus interactions and offer a foundation for improving the in vitro cultivation of Chinese cordyceps through targeted manipulation of microbial metabolites.

## Data Availability

The original contributions presented in this study are included in the article and Supplementary Materials. Further inquiries can be directed to the corresponding author.

## Supplementary Materials

The following supporting information can be downloaded at: https://www.mdpi.com/article/10.3390/jof11100746/s1, Figure S1: Kyoto Encyclopedia of Genes and Genomes (KEGG) pathway enrichment analysis of *Ophiocordyceps sinensis* following *Fusarium pseudonygamai* supernatant treatment; Figure S2: Validation of RNA-seq results by qRT-PCR; Figure S3: Effect of crude ethyl acetate extract from *Fusarium pseudonygamai* on *Ophiocordyceps sinensis* blastospore transformation; Figure S4: ^1^H and ^13^C NMR spectra of compounds isolated from the butanol crude extract of *F. pseudonygamai*; Figure S5: Effects of adenosine and benzoic acid on *Ophiocordyceps sinensis* blastospore transformation; Figure S6: Effect of mannitol biosynthesis modulators on the growth of *Fusarium pseudonygamai*; Supplementary Table S1: Primer’s sequence; Supplementary file S1: Differentially expressed genes (DEGs) in *Ophiocordyceps sinensis* following treatment with *Fusarium pseudonygamai* supernatant at day 4; Supplementary file S2: Gene Ontology (GO) enrichment analysis of differentially expressed genes in *Ophiocordyceps sinensis* following *Fusarium pseudonygamai* supernatant treatment at day 4; Supplementary file S3: Differentially expressed genes (DEGs) in *Ophiocordyceps sinensis* following treatment with *Fusarium pseudonygamai* supernatant at day 8; Supplementary file S4: Gene Ontology (GO) enrichment analysis of differentially expressed genes in *Ophiocordyceps sinensis* following *Fusarium pseudonygamai* supernatant treatment at day 8.

## References

[1] Lo H.C., Hsieh C., Lin F.Y., Hsu T.H. (2013). A systematic review of the mysterious caterpillar fungus *ophiocordyceps sinensis* in DongChongXiaCao (冬蟲夏草 dōng chóng xià cǎo) and related bioactive ingredients. J. Tradit. Complement. Med..

[2] Chen L., Teng H., Chen S., Zhou Y., Wan D., Shi Z. (2025). Future habitat shifts and economic implications for *Ophiocordyceps sinensis* under climate change. Ecol. Evol..

[3] Wei Y., Zhang L., Wang J., Wang W., Niyati N., Guo Y., Wang X. (2021). Chinese caterpillar fungus (*Ophiocordyceps sinensis*) in China: Current distribution, trading, and futures under climate change and overexploitation. Sci. Total Environ..

[4] Meng Q., Yu H.Y., Zhang H., Zhu W., Wang M.L., Zhang J.H., Zhou G., Li X., Qin Q., Hu S. (2015). Transcriptomic insight into the immune defenses in the ghost moth, *Hepialus xiaojinensis*, during an *Ophiocordyceps sinensis* fungal infection. Insect Biochem. Mol. Biol..

[5] Rao Z., Cao L., Wu H., Qiu X., Liu G., Han R. (2019). Comparative transcriptome analysis of *Thitarodes armoricanus* in response to the entomopathogenic fungi *Paecilomyces hepiali* and *Ophiocordyceps sinensis*. Insects.

[6] Li Y., Wang X., Jiao L., Jiang Y., Li H., Jiang S., Lhosumtseiring N., Fu S., Dong C., Zhan Y. (2011). A survey of the geographic distribution of *Ophiocordyceps sinensis*. Int. J. Microbiol..

[7] Winkler D. (2009). Caterpillar fungus (*Ophiocordyceps sinensis*) production and sustainability on the Tibetan Plateau and in the Himalayas. Asian Med..

[8] Liu G., Han R., Cao L. (2019). Artificial cultivation of the Chinese cordyceps from injected ghost moth larvae. Environ. Entomol..

[9] Cao L., Ye Y., Han R. (2015). Fruiting body production of the medicinal Chinese caterpillar mushroom, *Ophiocordyceps sinensis* (Ascomycetes), in artificial medium. Int. J. Med. Mushrooms.

[10] Qin Q.L., Zhou G.L., Zhang H., Meng Q., Zhang J.H., Wang H.T., Miao L., Li X. (2018). Obstacles and approaches in artificial cultivation of Chinese cordyceps. Mycology.

[11] Han R.C., Wu H., Tao H.P., Qiu X.H., Liu G.Q., Rao Z.C., Cao L. (2019). Research on Chinese cordyceps during the past 70 years in China. Chin. J. Appl. Entomol..

[12] Khalid M.Z., Khalid M.A., Han R., Cao L. (2024). The intricate dance: Exploring the interactions between entomopathogenic fungi and insects with special focus on the formation/production of Chinese cordyceps. Fungal Biol. Rev..

[13] Wu P., Qin Q., Zhang J., Zhang H., Li X., Wang H., Meng Q. (2022). The invasion process of the entomopathogenic fungus *Ophiocordyceps sinensis* into the larvae of ghost moths (*Thitarodes xiaojinensis*) using a GFP-labeled strain. Front. Microbiol..

[14] Liang Y., Hong Y., Mai Z., Zhu Q., Guo L. (2019). Internal and external microbial community of the *Thitarodes* moth, the host of *Ophiocordyceps sinensis*. Microorganisms.

[15] Wu H., Rao Z.C., Cao L., De Clercq P., Han R.C. (2020). Infection of *Ophiocordyceps sinensis* fungus causes dramatic changes in the microbiota of its *Thitarodes* host. Front. Microbiol..

[16] Chai W., Mao X., Li C., Zhu L., He Z., Wang B. (2024). Mannitol mediates the mummification behavior of *Thitarodes xiaojinensis* larvae infected with *Ophiocordyceps sinensis*. Front. Microbiol..

[17] Allocco J.J., Nare B., Myers R.W., Feiglin M., Schmatz D.M., Profous-Juchelka H. (2001). Nitrophenide (Megasul™) blocks *Eimeria tenella* development by inhibiting the mannitol cycle enzyme mannitol-1-phosphate dehydrogenase. J. Parasitol..

[18] Iwamoto K., Kawanobe H., Ikawa T., Shiraiwa Y. (2003). Characterization of salt-regulated mannitol-1-phosphate dehydrogenase in the red alga *Caloglossa continua*. Plant Physiol..

[19] Nguyen T., Kim T., Ta H.M., Yeo W.S., Choi J., Mizar P., Lee S.S., Bae T., Chaurasia A.K., Kim K.K. (2019). Targeting mannitol metabolism as an alternative antimicrobial strategy based on the structure-function study of mannitol-1-phosphate dehydrogenase in *Staphylococcus aureus*. mBio.

[20] Wang X., Li B., Liu D., Guo Y., Zhang J., Li W., Peng T., Ma Q., Shi X. (2024). Isolation, characterization, and LC MS/MS determination of anti-obesity components from pine needles of *Cedrus deodara* (Roxb.) G. Don. Front. Nutr..

[21] Nguyen P.H., Bui T.Q., Tran T.T., Bui T.T., Do T.T., To D.C., Tran M.H., Quy P.T., Co N.Q., Phu N.V. (2024). Inhibitory activities of *Aruncus dioicus* alkaloidal glycosides against protein tyrosine phosphatase 1B and α-glucosidase: A methodical theory-experiment investigation. Nat. Prod. Commun..

[22] Yi W., Chen C., Gan X. (2022). Active metabolites from the endophyte *Paenibacillus polymyxa* Y-1 of *Dendrobium nobile* for the control of rice bacterial diseases. Front. Chem..

[23] Yang R.H., Wang X.L., Su J.-H., Li Y., Jiang S.P., Gu F., Yao Y.-J. (2015). Bacterial diversity in native habitats of the medicinal fungus *Ophiocordyceps sinensis* on Tibetan Plateau as determined using Illumina sequencing data. FEMS Microbiol. Lett..

[24] Wang Z., Gerstein M., Snyder M. (2009). RNA-Seq: A revolutionary tool for transcriptomics. Nat. Rev. Genet..

[25] Yin H., Duo H., Li S., Qin D., Xie L., Xiao Y., Sun J., Tao J., Zhang X., Li Y. (2024). Unlocking biological insights from differentially expressed Genes: Concepts, methods, and future perspectives. J. Adv. Res..

[26] Sellem C.H., Marsy S., Boivin A., Lemaire C., Sainsard-Chanet A. (2007). A mutation in the gene encoding cytochrome c1 leads to a decreased ROS content and to a long-lived phenotype in the filamentous fungus *Podospora anserina*. Fungal Genet. Biol..

[27] Zeng G., Xu X., Kok Y.J., Deng F.S., Chow E.W.L., Gao J., Bi X., Wang Y. (2023). Cytochrome c regulates hyphal morphogenesis by interfering with cAMP-PKA signaling in *Candida albicans*. Cell Rep..

[28] Turrion-Gomez J., Eslava A., Benito E. (2010). The flavohemoglobin BCFHG1 is the main NO detoxification system and confers protection against nitrosative conditions but is not a virulence factor in the fungal necrotroph *Botrytis cinerea*. Fungal Genet. Biol..

[29] Jürgensen C.W., Jacobsen N.R., Emri T., Havn Eriksen S., Pócsi I. (2001). Glutathione metabolism and dimorphism in *Aureobasidium pullulans*. J. Basic Microbiol..

[30] Thomas D., Klein K., Manavathu E., Dimmock J., Mutus B. (1991). Glutathione levels during thermal induction of the yeast-to-mycelial transition in *Candida albicans*. FEMS Microbiol. Lett..

[31] Manavathu M., Manavathu E., Gunasekaran S., Porte Q., Gunasekaran M. (1996). Changes in glutathione metabolic enzymes during yeast-to-mycelium conversion of *Candida albicans*. Can. J. Microbiol..

[32] Pócsi I., Molnár Z., Pusztahelyi T., Varecza Z., Emri T. (2007). Yeast-like cell formation and glutathione metabolism in autolysing cultures of *Penicillium chrysogenum*. Acta Biol. Hung..

[33] Wangsanut T., Pongpom M. (2022). The role of the glutathione system in stress adaptation, morphogenesis and virulence of pathogenic fungi. Int. J. Mol. Sci..

[34] Polekhina G., Board P., Blackburn A., Parker M. (2001). Crystal structure of maleylacetoacetate isomerase/glutathione transferase zeta reveals the molecular basis for its remarkable catalytic promiscuity. Biochemistry.

[35] Vélëz H., Glassbrook N.J., Daub M.E. (2007). Mannitol metabolism in the phytopathogenic fungus *Alternaria alternata*. Fungal Genet. Biol..

