# Supplementary material for: Fusarium pseudonygamai Promotes Blastospore Transformation in Ophiocordyceps sinensis: Insights into Microbial Interaction and Key Mechanisms
Source: J Fungi (Basel). 2025 Oct 18;11(10):746. doi: 10.3390/jof11100746 (PMC12565527; doi:10.3390/jof11100746)
Supplement: Supplementary file 1 [file jof-11-00746-s001.zip › Supplementary figures and table S1.pdf]

Supplementary figures:

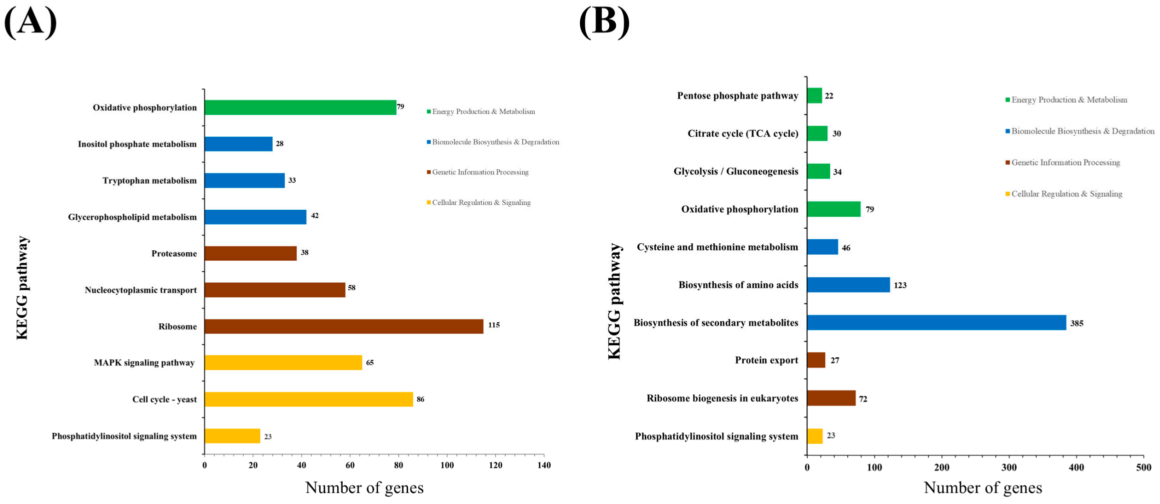

Figure S1. Kyoto Encyclopedia of Genes and Genomes (KEGG) pathway enrichment analysis of *Ophiocordyceps sinensis* following *Fusarium pseudonygamai* supernatant treatment. (**A-B**) Significantly enriched pathways at (**A**) day 4 and (**B**) day 8.

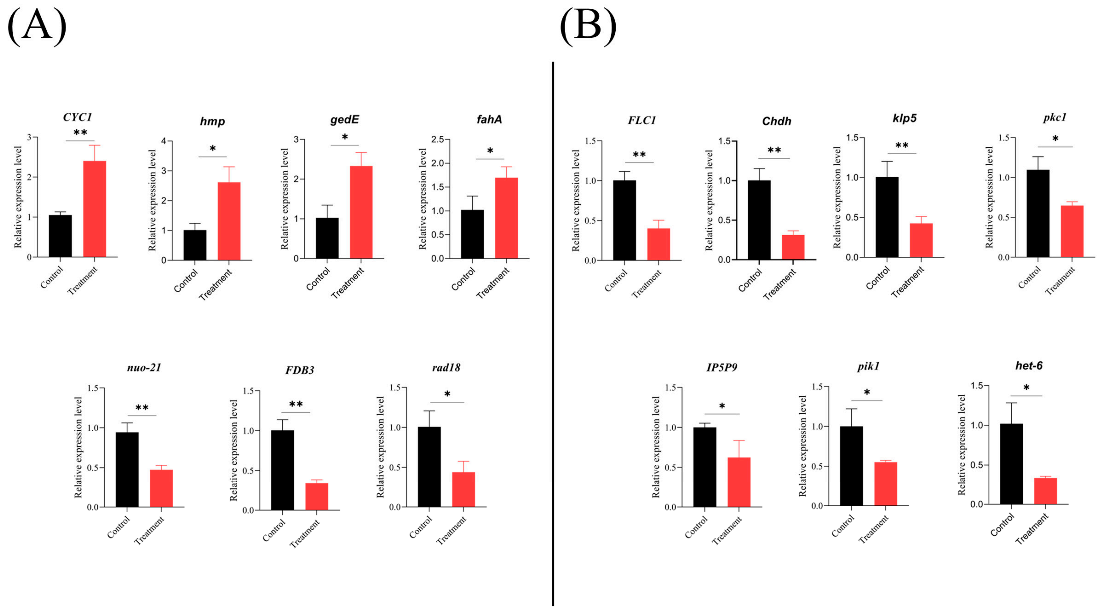

Figure S2. Validation of RNA-seq results by qRT-PCR. (**A-B**) Relative mRNA expression levels of selected genes in *Ophiocordyceps sinensis* blastospores treated with *Fusarium pseudonygamai* supernatant at **(A)** day 4 and **(B)** day 8. Expression levels are normalized to the reference gene *TUBB* and shown relative to the control group. Data are presented as mean ± SD and analyzed using a two-tailed unpaired Student's t-test (*$p < 0.05$, **$p < 0.01$).

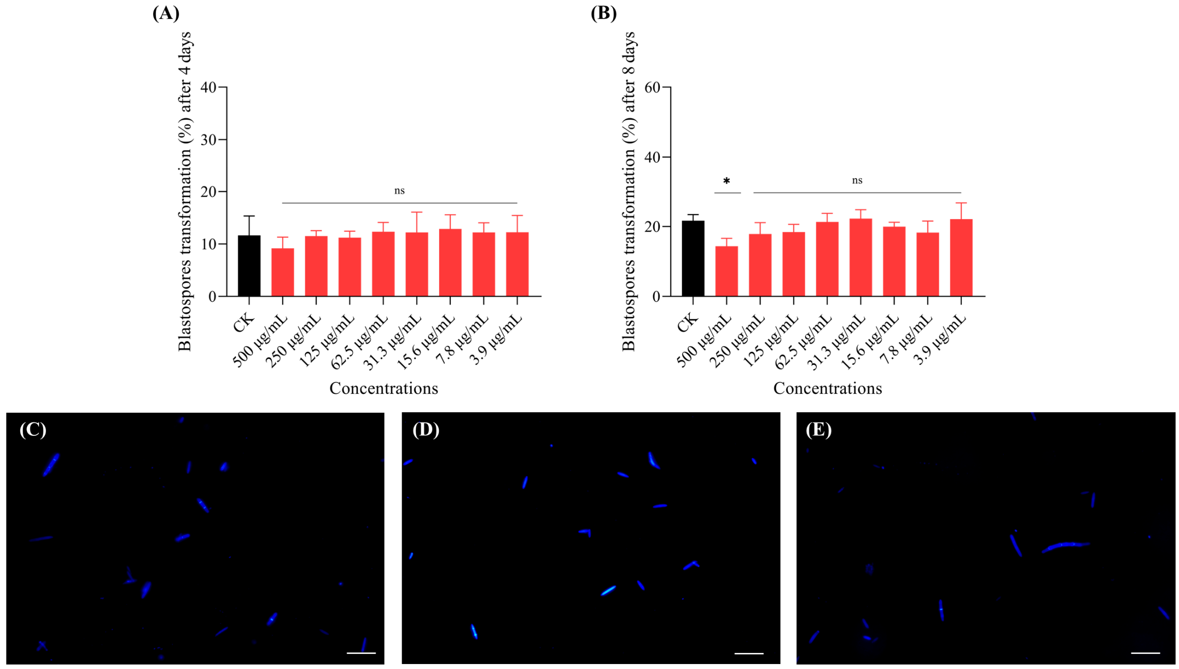

Figure S3. Effect of crude ethyl acetate extract from *Fusarium pseudonygamai* on *Ophiocordyceps sinensis* blastospore transformation. **(A–B)** Bar graphs showing the effect of ethyl acetate crude extract on *O. sinensis* transformation at **(A)** day 4 and **(B)** day 8. **(C)** Confocal microscopic image of the control group (CK) at day 8. **(D)** Confocal microscopic image of blastospores treated with 500 µg/mL ethyl acetate extract at day 8. **(E)** Confocal microscopic image of blastospores treated with 15.6 µg/mL ethyl acetate extract at day 8. Scale bar 50 µm. Data are presented as mean ± SD and analyzed using one-way ANOVA followed by Dunnett's post hoc test (*$p < 0.05$, ns = not significant).

(A)

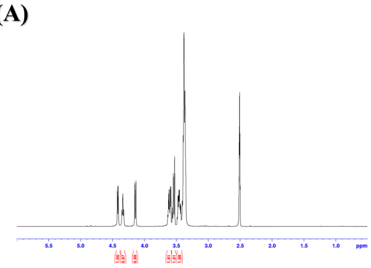

^{1}H spectrum of Mannitol (FB30)

(C)

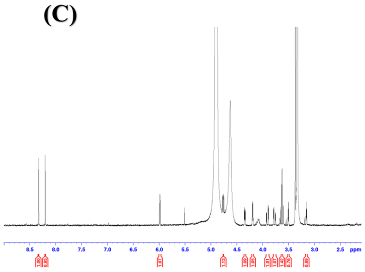

^{1}H spectrum of Adenosine (FB25-27)

(E)

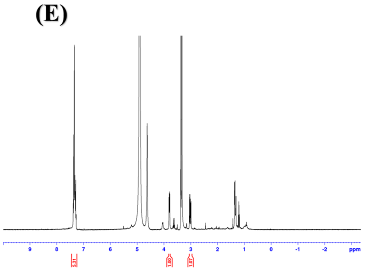

^{1}H spectrum of Benzoic acid (FB30-38gi11-12)

(B)

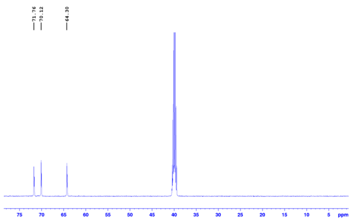

^{13}C spectrum of Mannitol (FB30)

(D)

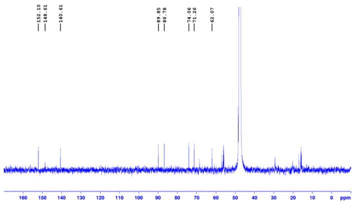

^{13}C spectrum of Adenosine (FB25-27)

(F)

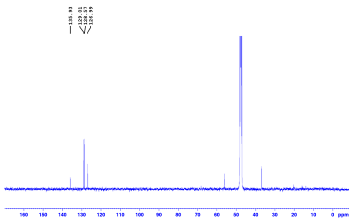

^{13}C spectrum of Benzoic acid (FB30-38gi11-12)

Figure S4. ^{1}H and ^{13}C NMR spectra of compounds isolated from the butanol crude extract of *F. pseudonygamai*. (A-B) Spectra of Mannitol (FB30), (C-D) Adenosine (FB25-27), (E-F) Benzoic acid (FB30-38gi11-12).

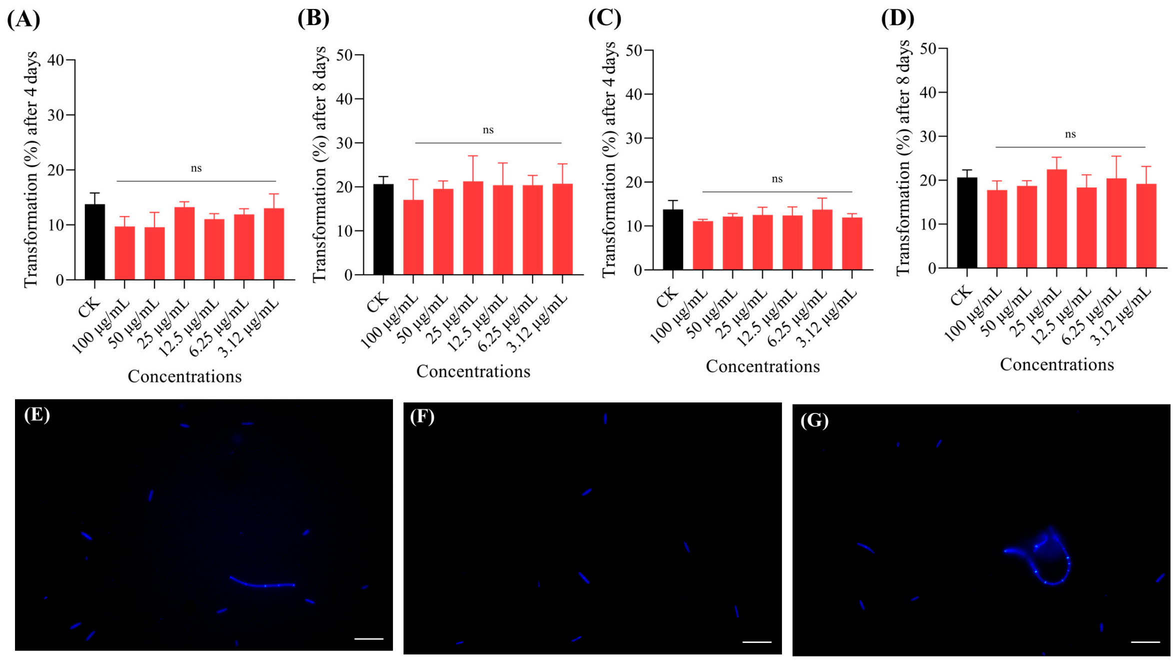

Figure S5. Effects of adenosine and benzoic acid on *Ophiocordyceps sinensis* blastospore transformation. Bar graphs showing the effect of adenosine on *O. sinensis* blastospore transformation at (**A**) day 4 and (**B**) day 8. Bar graphs showing the effect of benzoic acid on *O. sinensis* blastospore transformation at (**C**) day 4 and (**D**) day 8. (**E**) Confocal microscopic images from control (CK) group at day 8. (**F**) Confocal microscopic images from adenosine treated group (50 μg/mL) at day 8. (**G**) Confocal microscopic images from benzoic acid treated group (50 μg/mL) at day 8. Scale bar 50 μm. Data are presented as mean ± SD and analyzed using one-way ANOVA followed by Dunnett's post hoc test (ns = not significant, $p > 0.05$).

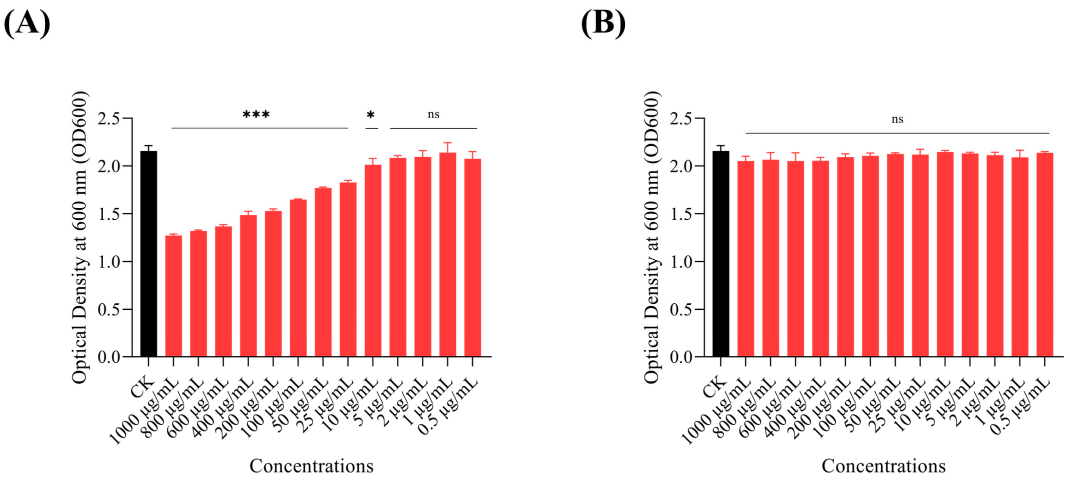

Figure S6. Effect of mannitol biosynthesis modulators on the growth of *Fusarium pseudonygamai*. (A) Bar graph showing the effect of different concentrations of 3-nitrophenyl disulfide on *F. pseudonygamai* growth after 4 days. (B) Bar graph showing the effect of different concentrations of dihydrocelastrol on *F. pseudonygamai* growth after 4 days. Growth was measured by optical density at 600 nm (OD_{600}) after 4 days of treatment. Data are presented as mean ± SD and analyzed using one-way ANOVA followed by Dunnett's post hoc test (*$p < 0.05$, ***$p < 0.001$, ns = not significant).

Supplementary Table S1: Primer's sequence

Name	Primer Sequences (*5'-3'*)
Cyc1	CACCGACGCCAACAAGCAAA
	CTTCTTCAGGCCGCCAAAGG
Hmp	TGGATGGTGCAGGCCAGGAA
	CCCGTGCTAAACAGCTCGACAAAG
gedE	GGCGTCAGCCTCGAAGAGTT
	TGTCGTTGTCAAATGCGGTGT
fahA	ACGACACGCCTCTTCTGCCCTAT
	GGTTGCAGCCGCTAATGGTG
FDB3	GAAGCGTCCGTGAGGCAGTT
	GGATGAGCCCATGACAAGCAGT
nuo-21	GCTGGCAATCATCTGGAGAC
	GAATAAAGAAAGTTGTTGGCGTAG
rad18	TCACATCGTGCTCGCATACTTT
	ATTCGACCGCTTCCTCAACC
IP5P9	GACACGGCGGTCAAGGATGG
	GCGTAAAGACGGACACGATTGG
pkc1	TTGCGGGACGCCAGAGTTTA
	AATCGGGTACAGCGGTTCGT
pik1	TCTCGGCGACTGTTTGATTTG
	TCTGGACCTGTCTTGCGTTGTAT
FLC1	GCGTCGTCCTGATTGTCGTTC
	CATTTCCTTTCTGCGCTTTCG
Chdh	GGACGCCAAGTTCACCGAGGAG
	TCGTGTTTGGAGCCAATGGGATT
het-6	GCTCCCGCCGTCGTCTTTGT
	GCCCAGTGCGTGCTGTAGATGTT
Klp5	GCAAGCAGTGCGACAAGAGGG
	CGTCAAAGGCGGCAATCCAG
M1PDH	ACATACTCACAATCCCACTTACAC
	GTTGACATCAGCGAAGACGA
MDH	GGCTTCCTCTACGCTGCTCT
	GGTGATGGTTCCGTTCTTCTG
TUBB	GCGTCGTCCTGATTGTCGTTC
	CATTTCCTTTCTGCGCTTTCG
